# A Complete Digital Workflow Using the Additive Manufacturing Injection Molding (AMIM) Technique for Polychromatic Anterior Resin Composite Restorations

**DOI:** 10.7759/cureus.82989

**Published:** 2025-04-25

**Authors:** Leonardo M Nassani, Rafat S Amer, Shereen S Azer, Daniel S Clark, Khalil Ezzeddine

**Affiliations:** 1 Division of Restorative and Prosthetic Dentistry, The Ohio State University College of Dentistry, Columbus, USA; 2 Department of Dentistry, Dental Art Studio Inc., Laval, CAN

**Keywords:** additive manufacturing, aesthetic dentistry, injection molding, polychromatic resin composite, resin composite, restorative digital workflow

## Abstract

This case report introduces the Additive Manufacturing Injection Molding (AMIM) technique, a fully digital workflow using orthodontic 3D-printed resin for polychromatic anterior composite restorations. The technique aims to improve precision and efficiency compared to traditional methods. This report demonstrates the feasibility and predictability of using flexible 3D-printed resin in conjunction with a digital workflow to achieve aesthetically pleasing outcomes in a patient seeking treatment for short lateral incisors and hypocalcification staining.

## Introduction

The pursuit of aesthetic excellence in dental restorations remains a cornerstone of modern restorative dentistry. Patients increasingly demand natural-looking outcomes that seamlessly blend with their dentition, necessitating restorations that replicate the morphology, polychromaticity, translucency, and texture of natural teeth [1]. Traditional resin composite layering techniques, while effective, are extremely skill-dependent, very time-consuming, and prone to variability [2,3].

To reduce variability and chair time, a technique involving injection molding direct composite resin veneers has been proposed. This conventional method involves creating an ideal smile wax-up, which is subsequently duplicated using a clear matrix made of translucent vinyl polysiloxane (VPS) for the injection of composite material. While effective, this process is not without its limitations. Preoperative lab work is time- and material-intensive, and the outcome lacks predictability [4]. 

The advent of intraoral scanners, computer-aided design (CAD), and computer-aided manufacturing (CAM) technologies has revolutionized traditional dental practices [5,6].

Moreover, recent advancements in additive manufacturing (AM) and dental materials offer transformative potential to address these challenges. This paper introduces a novel complete digital workflow integrating Additive Manufacturing Injection Molding (AMIM), a hybrid technique combining the precision of AM with the efficiency of injection molding, to fabricate direct polychromatic anterior restorations. By utilizing digital design and 3D-printed molds, AMIM aims to standardize aesthetic outcomes while reducing manual intervention.

A notable example of dental material advancement is the recently introduced biocompatible flexible 3D-printed resins [7]. These flexible 3D-printed resins are used in indirect bonding techniques for orthodontic bracket placement. This digital approach not only enhances precision and efficiency but also offers customized solutions tailored to individual patients, thereby demonstrating high reliability in clinical settings.

Another advancement in dental materials that makes the AMIM technique possible is injectable, highly filled composite resins. We used the G-ænial Universal Injectable (GC Corporation, Tokyo, Japan) composite resin in this case report. This material offers excellent strength and gloss retention, maximizing restoration longevity, and is available in 15 shades and three levels of translucency [8]. 

It is important to differentiate these materials from flowable composites. While both are injectable, highly filled composites have a higher viscosity and filler content, resulting in superior mechanical properties and wear resistance.

This case report aims to bridge the gap between traditional polychromatic restorative techniques and digital innovations. We specifically leverage the use of orthodontic 3D-printed resins in anterior composite restorations, introducing a novel technique: AMIM.

## Case presentation

**Figure 1:**
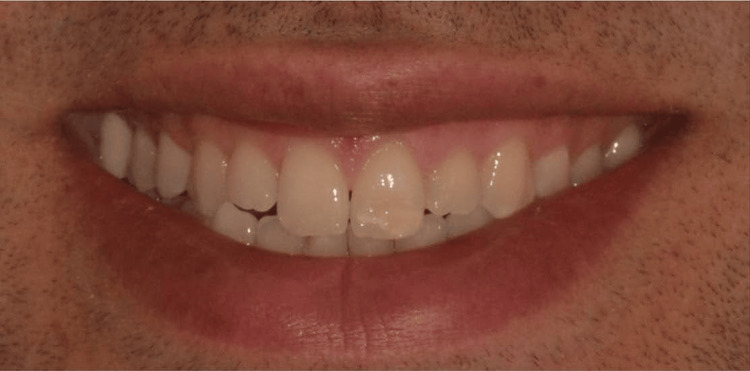
Preoperative photograph.

The patient, a 28-year-old man, presented with aesthetic complaints regarding short maxillary lateral incisors and hypocalcification staining on the left maxillary central incisor (Figure 1). While unhappy with the appearance of his smile, he wished to preserve healthy tooth structure. 

**Figure 2:**
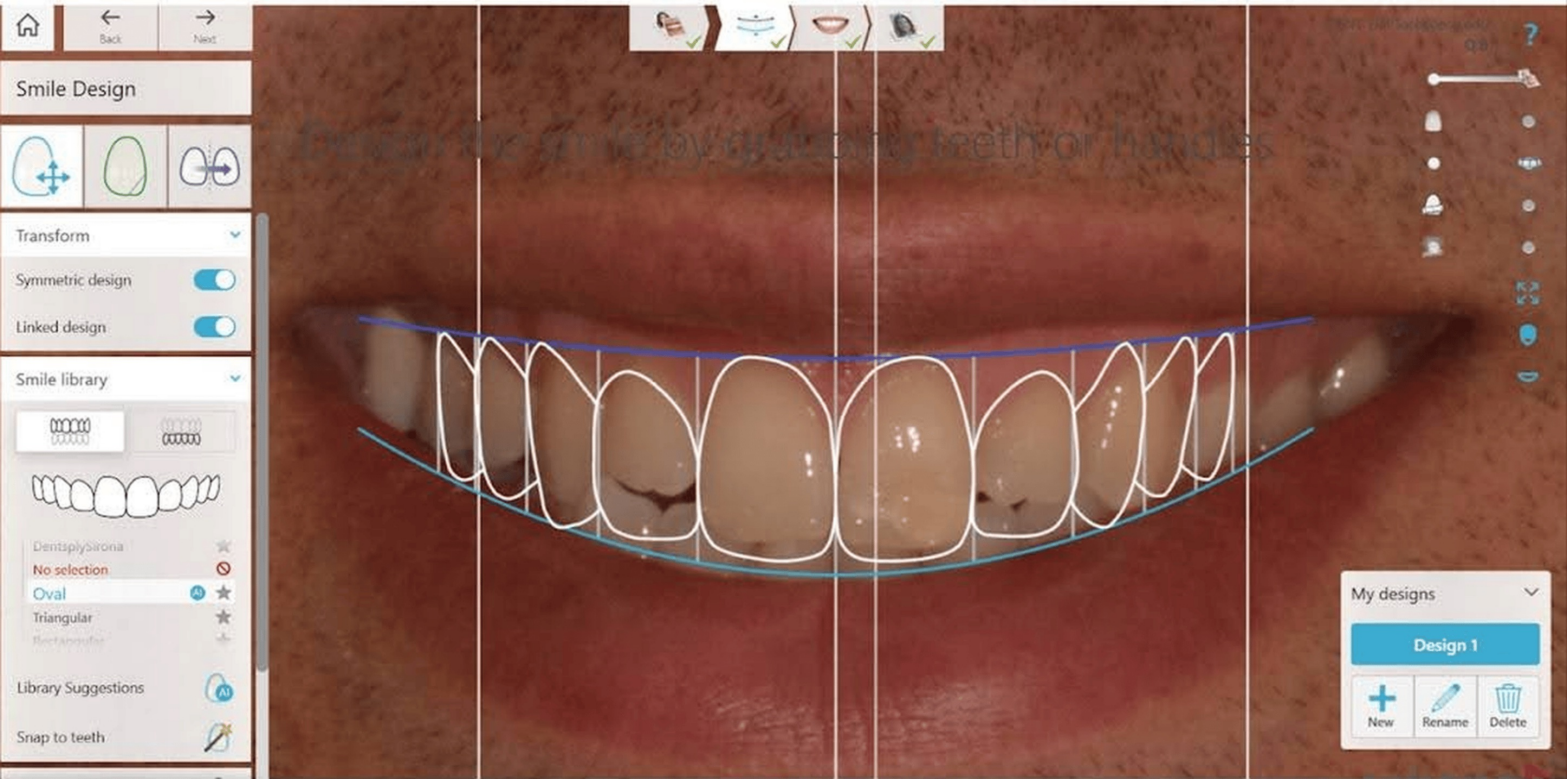
Digital smile design.

At the first appointment, photographs were taken with a DSLR camera (Nikon D3100, Nikon, Tokyo, Japan). These photos were used for digital smile design [9,10] using the 3Shape Dental System software (Version 2021, 3Shape, Copenhagen, Denmark) (Figure 2). The patient's dentition was digitally scanned using the Trios 4 intraoral scanner (3Shape, Copenhagen, Denmark). 

After discussing various treatment options, including porcelain laminate veneers and direct composite resin veneers, the patient chose no-prep direct composite veneers [11].

A treatment plan was developed for anterior veneers from the right maxillary canine to the left maxillary canine to address the short lateral incisors and mask the existing hypocalcification stain, utilizing a complete digital workflow through the AMIM technique for anterior resin composite restorations. 

**Figure 3:**
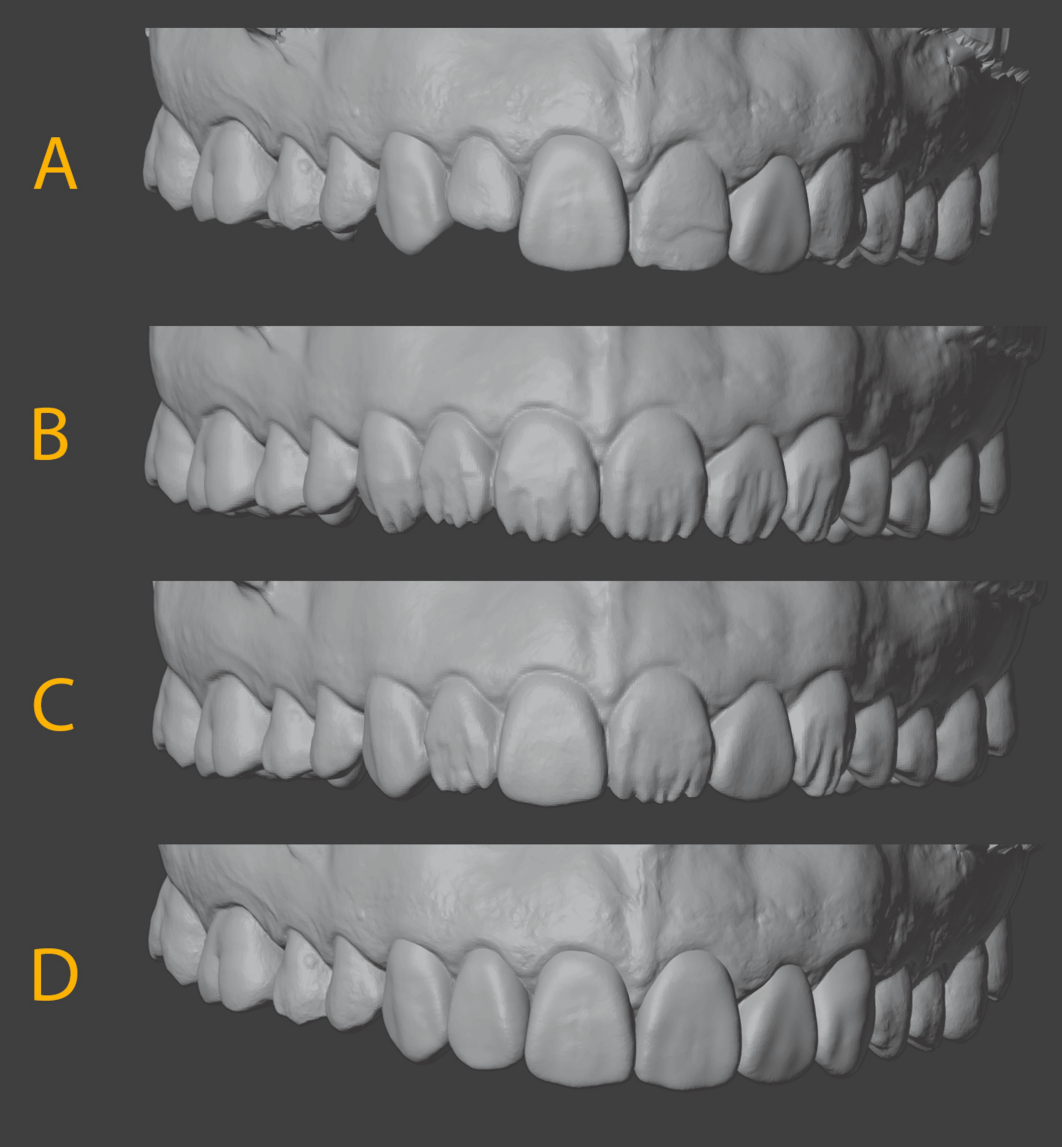
(A) Diagnostic intraoral scan. (B) Digital design for dentin layer for all teeth. (C) Digital design for enamel layer for the maxillary right canine, right central incisor, and left lateral incisor. (D) Digital design for all teeth to be treated.

Using the intraoral scan, three virtual models were created using the 3Shape Dental System software. The first model has the maxillary right canine through the maxillary left canine modeled to reflect the designed dentin layer. The second model created the enamel layer and overall tooth shape for the maxillary right canine, right central incisor, and left lateral incisor. The third model has the final desired aesthetics of all teeth (Figure 3).

**Figure 4:**
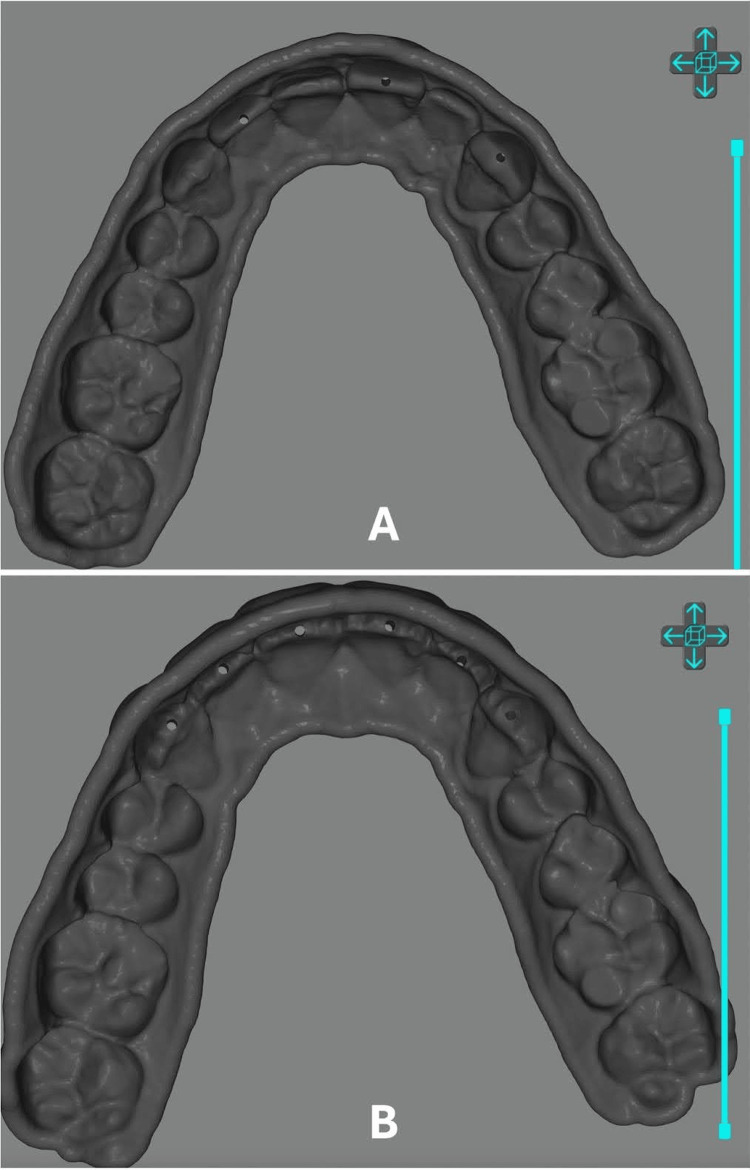
(A) Guide 2 designed from the alternating model with injection channels in the incisal third. (B) Guide 3 designed with all modeled teeth with injection channels.

Three virtual injection molding guides were made based on the digital models using the 3Shape Dental System Splint Studio covering 1 mm of the soft tissue at a thickness of 1.5 mm and undercuts blocked out. These injection guides were exported as Standard Tessellation Language (STL) files from the 3Shape software and imported in the Anycubic Workshop (Version 2.1.29, Anycubic, Shenzhen, China) for the placement of 1 mm incisal channels on all modeled maxillary teeth for the first guide (guide 1) and for the right canine, right central incisor, and left lateral incisor for the second guide (guide 2). For the third guide (guide 3), these incisal channels were placed for the remaining teeth to be veneered. These channels will allow the injectable composite to flow around the tooth and form the veneer (Figure 4).

**Figure 5:**
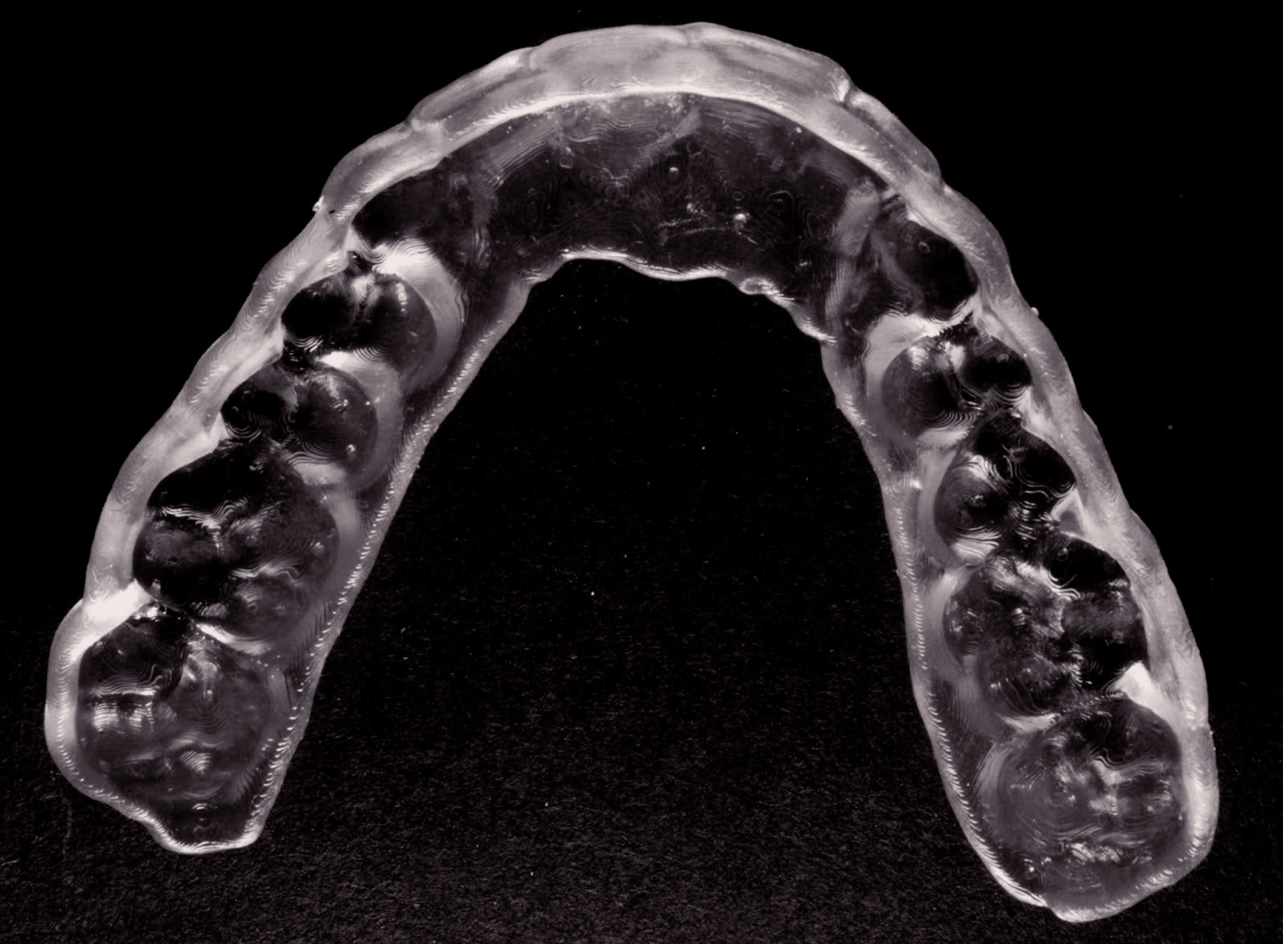
3D-printed injection molding guide using Formlabs IBT Flex Resin for composite resin injection.

The injection molding guides were 3D-printed with a Formlabs Form 3B+ printer (Formlabs, Somerville, Massachusetts, United States), using Formlabs IBT Flex Resin. This resin was selected for its translucency and flexibility [12], allowing for the efficient injection of composite resin (Figure 5).

After printing, the injection guides were verified for fit and adaptation. DuPont Non-stick Dry-Film spray (DuPont, Wilmington, Delaware, United States) was applied to the intaglio surface of the injection guides to enhance the ease of matrix removal and prevent adhesion of the injectable composite material to the 3D-printed guides. This treatment ensured smooth separation of the guides from the cured resin matrix, minimizing the risk of distortion and ensuring the precision of the final restoration.

No tooth preparation was performed, and no local anesthetic was administered. A dental prophylaxis procedure was performed on all teeth to be treated utilizing a pumice-based abrasive agent and rotary prophylaxis cups to achieve supragingival plaque removal and enamel surface polishing. Isolation was achieved using OptraGate (Ivoclar, Schaan, Liechtenstein) and cotton rolls. 

Enamel surface etching using an etching gel containing 37% phosphoric acid (Ivoclar, Schaan, Liechtenstein) was applied to the maxillary right canine, right lateral and central incisors, left central and lateral incisors, and left canine for 15 seconds. Etchant was rinsed, and the teeth were dried. The bonding system, Adhese Universal VivaPen (Ivoclar, Schaan, Liechtenstein), was applied to the etched surfaces and light-cured according to the manufacturer's instructions using Coltolux LED curing light (Coltene, Cuyahoga Falls, Ohio, United States). 

**Figure 6:**
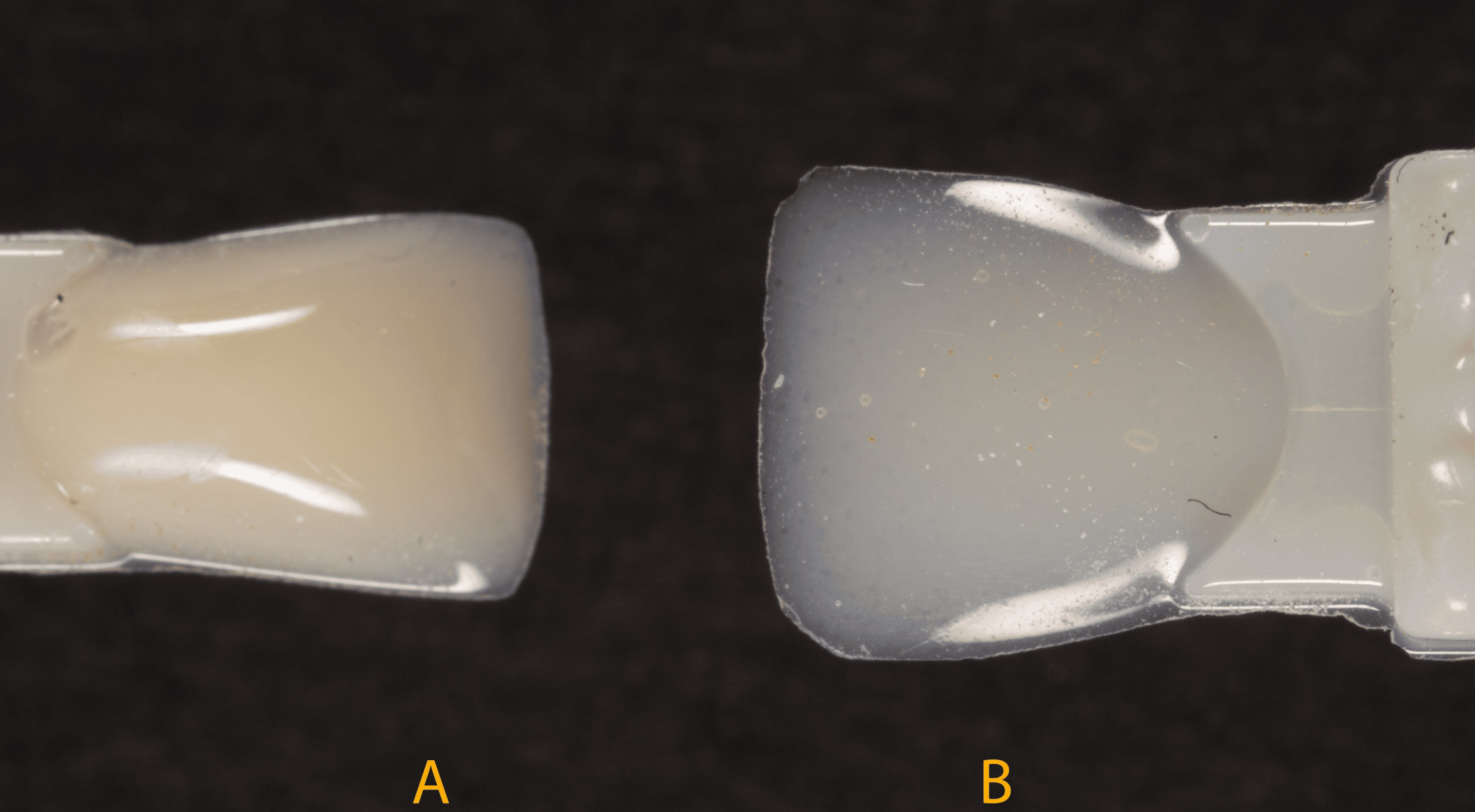
(A) Shade AO1 for the inner dentin layer. (B) Shade JE for the outer enamel layer.

Guide 1 was placed in position intraorally, and the G-ænial Universal Injectable composite resin shade AO1 was injected into the guide (Figure 6). 

**Figure 7:**
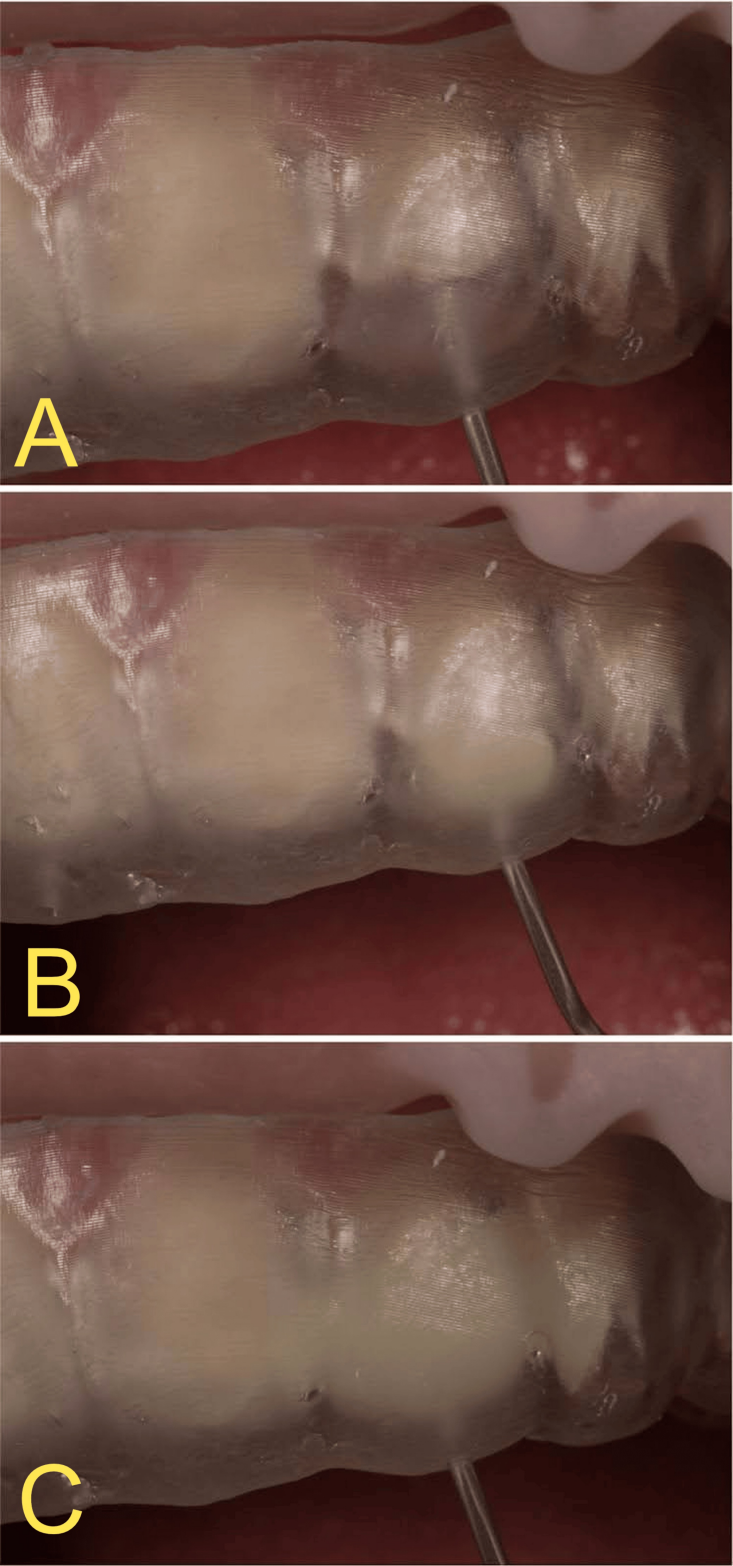
Injection of the G-ænial Universal Injectable composite resin into the guide and forming the veneer on the maxillary left lateral incisor.

The process of injecting the composite was done by inserting the pointed tip of the composite syringe through the previously placed holes at the incisal third of the molded teeth and slowly injecting the composite until the dentin layer veneer was formed around the tooth (Figure 7).

**Figure 8:**
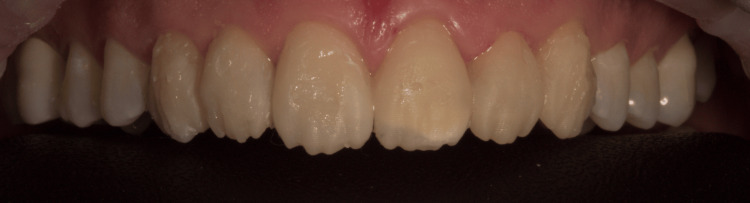
Dentin layer veneer after injection with the first guide.

The composite syringe was removed from the guide, and tack-curing was done by placing LED curing light close to each tooth and shining the light through the translucent guide for three seconds for each tooth before carefully removing it. Excess material in the interproximal sulcus was carefully removed using a number 12 scalpel blade. Final polymerization was achieved by directly shining light on each surface for 20 seconds. This created the opaque dentin layer of the veneers (Figure 8). 

To achieve the polychromatic effect and to layer the translucent enamel layer over the opaque dentin layer, the second injection guide was placed after isolating the maxillary right lateral incisor, left central incisor, and left canine with polytetrafluoroethylene (PTFE) tape and injecting with translucent composite shade JE around the maxillary right canine, right central incisor, and left lateral incisor, as previously described.

After complete polymerization of this step, the third injection guide was placed, and the process was repeated to restore the remaining teeth. The restorations were contoured using fine flame diamond burs (Brasseler, Savannah, Georgia, United States), occlusion was checked and adjusted, and the restorations were polished using the Sof-Lex polishing system (3M ESPE, St. Paul, Minnesota, United States).

**Figure 9:**
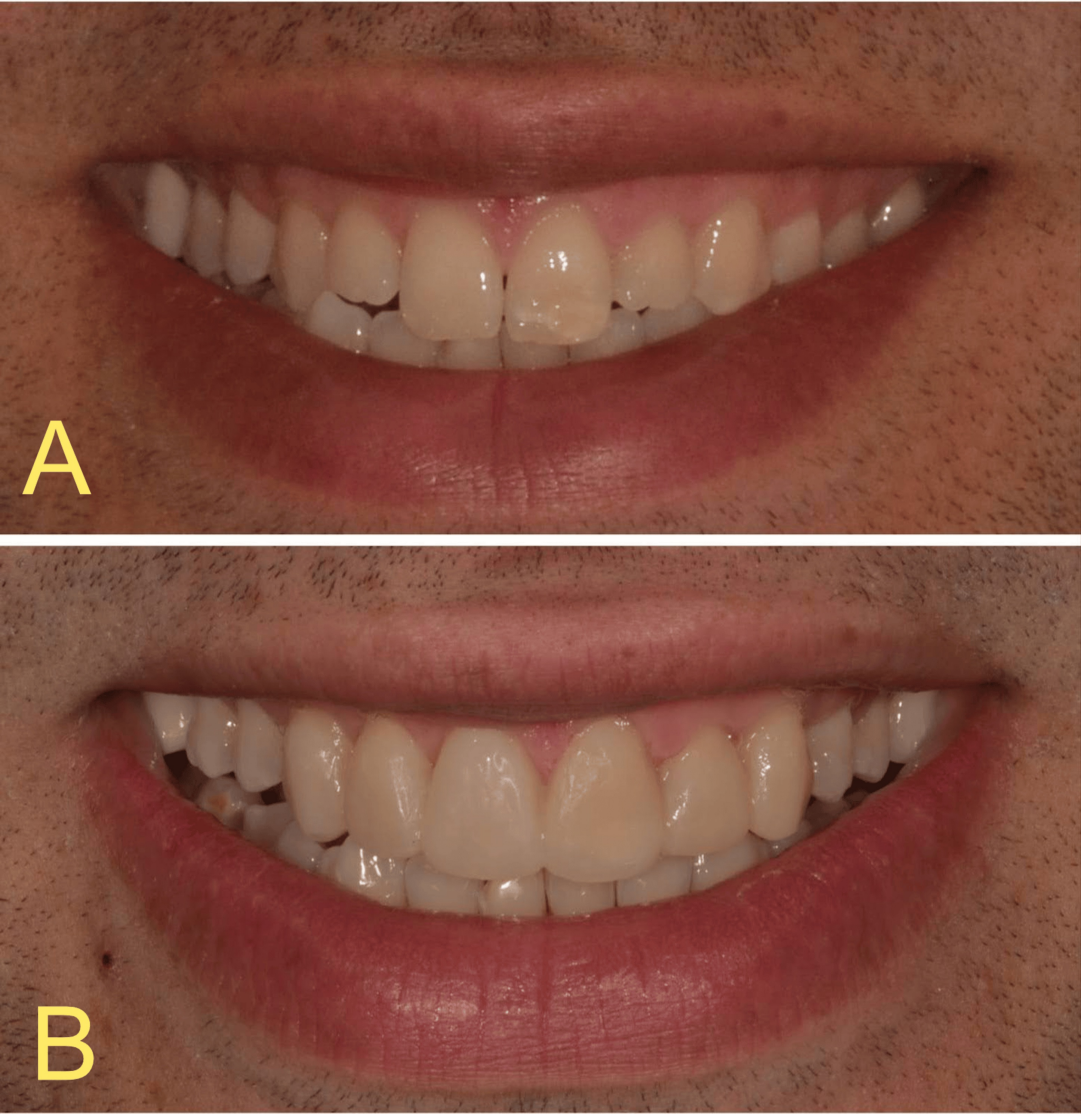
(A) Before and (B) immediately after the case was completed with the Additive Manufacturing Injection Molding direct resin composite veneer technique.

Figure 9 shows immediate postoperative results.

## Discussion

Direct veneers were chosen over indirect veneers because of their less invasive nature. The procedure is simpler and, in the presented case, required no tooth preparation or anesthesia.

The success of the AMIM technique hinges on the unique properties of IBT Flex Resin. It is a biocompatible 3D printing resin, and its flexibility allows for the safe removal of the molding matrix without distortion, a common problem with traditional rigid matrices [13]. This flexibility also allows the procedure to be performed without tooth preparation, as undercuts do not need to be addressed. IBT Flex is also translucent, ensuring adequate light transmission during the curing process. This is crucial for achieving the optimal mechanical properties of the composite resin, such as strength, wear resistance, and longevity.

These include the high-resolution printing capabilities with the Formlabs printer, allowing for detailed and accurate restorations and resistance to tearing when flexed, ensuring the integrity of the molding matrix during removal.

By eliminating manual steps like wax-ups and silicone matrix fabrication, the fully digital workflow reduces variability and potential errors, resulting in predictable, high-quality restorations [14].

While the digital workflow offers numerous advantages, cost and accessibility to 3D printing equipment can be barriers in clinical settings [15]. Long-term studies are also necessary to evaluate the durability and clinical performance of restorations created using AMIM. Finally, the technique has a learning curve, and achieving ideal interproximal contact can be challenging. Clinicians will require adequate training to implement the digital workflow effectively.

This case explored the application of orthodontic 3D-printed IBT Resin in anterior composite restorations, introducing the AMIM technique for direct polychromatic composite veneers. The technique was successfully implemented in a patient case, demonstrating its feasibility and potential for improving precision and efficiency in restorative dentistry.

## Conclusions

Orthodontic 3D-printed indirect bonding tray resin can be adapted for restorative dentistry due to its clear and flexible properties, and a complete digital workflow for anterior composite resin injection molding using the AMIM technique is feasible, reliable, and predictable. The integration of digital scanning, CAD, and 3D printing resulted in precise, efficient, and predictable outcomes for treating short maxillary laterals and hypocalcification staining. This approach provides a streamlined solution, enhancing precision and efficiency in anterior composite resin restorations while opening opportunities for advanced applications in restorative dentistry.
